# Transoral Robotic Surgery (TORS) in Head and Neck Reconstruction

**DOI:** 10.3390/jcm14165775

**Published:** 2025-08-15

**Authors:** Sophia Sijia Song, Z-Hye Lee, Jessie Zexi Yu

**Affiliations:** 1 Albany Medical College, Albany, NY 12208, USA; songs1@amc.edu; 2 UT MD Anderson Cancer Center, Houston, TX 77030, USA; zlee@mdanderson.org

**Keywords:** transoral robotic surgery, TORS, head and neck reconstruction, robotic-assisted reconstruction

## Abstract

**Background/Objectives:** Transoral robotic surgery (TORS) has advanced treatment for complex oropharyngeal cancers (OPC) by offering minimally invasive, precise approaches, initially for ablative and now for reconstructive procedures. This review examines TORS in OPC reconstruction, comparing it to traditional methods and presenting a TORS case with free flap reconstruction in recurrent OPC. **Methods:** A PubMed review assessed TORS-based reconstruction outcomes, technical challenges, and innovations. Additionally, a recurrent OPC case was treated with TORS resection and lateral arm free flap. Surgical techniques and outcomes were detailed. **Results:** TORS is associated with reduced morbidity and length of hospitalization, and showed positive functional outcomes in primary, salvage, and post-radiation cases. Our case achieved favorable oncologic and functional outcomes, such as preserved speech and swallowing. **Conclusions:** TORS-based reconstruction represents a major advancement in minimally invasive head and neck oncology, providing comparable oncologic outcomes to open surgery with fewer complications. Although technically demanding, TORS is promising for complex reconstructions, warranting further research to refine techniques and standardize protocols.

## 1. Introduction

Transoral Robotic Surgery (TORS) has revolutionized head and neck surgery by providing a minimally invasive alternative to traditional open approaches for tumor resection. Since its introduction, TORS has rapidly gained popularity due to its ability to provide excellent three-dimensional visualization, precise surgical control, and access to anatomically challenging regions. Initially, TORS was primarily used for smaller tumors that did not require reconstruction, allowing these cases to heal by secondary intention [1]. However, as surgeon expertise and technological advancements have progressed, TORS has expanded to include larger resections, often requiring complex reconstructive procedures, particularly in the setting of salvage surgery [2].

The application of TORS is particularly significant in oropharyngeal cancer (OPC), which is the most common mucosal malignancy in the head and neck region. OPC typically affects structures such as the soft palate, tonsils, base of the tongue, and pharyngeal walls, and over 70% of new cases are linked to human papillomavirus (HPV) infection, predominantly occurring in younger, healthier, non-smoking patients [3]. The treatment of OPC traditionally involved highly invasive surgical techniques, such as lip-splitting mandibulotomy and tongue release, which often led to significant complications, including mandibular malunion, scarring, osteoradionecrosis, and long-term dysphagia [4]. These morbidities greatly impacted patient recovery and quality of life, leading to a search for less invasive alternatives.

TORS, with its ability to achieve precise resections and minimize trauma, emerged as an ideal solution for addressing these challenges. Studies like that of O’Malley et al. demonstrated the advantages of TORS in treating hard-to-reach areas, such as the base of the tongue [5]. Their work highlighted the ability of robotic surgery to preserve key anatomical structures while achieving negative surgical margins with minimal complications. As such, TORS has gradually replaced more invasive procedures, improving morbidity while maintaining oncologic effectiveness.

As TORS evolved, its role expanded beyond tumor ablation to include reconstruction, particularly in complex head and neck surgeries. The ability to combine minimally invasive resection with precise, robotic-assisted reconstruction has led to reduced patient morbidity, quicker recovery, and improved overall outcomes [6]. In salvage cases, TORS reconstruction has shown promising results compared to the traditional open surgery approach [2]. However, reconstruction after TORS presents unique challenges due to limited access for flap inset and microvascular anastomosis. For example, Arora et al. discussed the technical difficulties of free flap reconstruction following TORS, emphasizing the need for specialized modifications to ensure successful outcomes [7]. Despite these challenges, TORS offers several advantages in reconstruction, including improved visualization and enhanced precision for flap inset, which are critical in achieving optimal functional and aesthetic outcomes [8].

The increasing application of TORS in head and neck oncology has also fueled the growth of robot-assisted reconstruction (RAR). Chalmers et al. outlined the expanding role of TORS in larger, multi-site resections, which often require complex reconstructions involving free-flap techniques [9]. In these cases, RAR has emerged as a vital tool, enabling surgeons to address intricate defects that extend beyond the oropharynx, improving both oncologic outcomes and patient recovery.

The growing adoption of TORS for head and neck cancer, particularly in oropharyngeal cancer, has not only transformed the approach to tumor resection but has also led to the development of innovative reconstructive techniques. As TORS continues to evolve, its applications in both primary and salvage surgeries are expanding, offering a less invasive yet highly effective option for managing complex head and neck cancers. This literature review will discuss the expansion of TORS applications and indications, compare it with traditional surgical approaches, outline the reconstructive classification and techniques, and assess the oncologic and functional outcomes of TORS reconstruction, as well as addressing the associated challenges and limitations. Further, a case report will detail flap design and harvest, flap inset technique and microsurgical anastomosis, and the patient’s postoperative course. Relevant studies were identified through a comprehensive PubMed search using associated keywords.

## 2. Review of TORS-Based Reconstruction in Head and Neck Oncology

### 2.1. Expansion of TORS Applications and Indications

TORS has expanded significantly in its applications for head and neck cancer, particularly in OPC. Originally approved by the FDA for early-stage T1 and T2 tumors, TORS is now utilized in select T3 cases such as those involving exophytic tumors and unlikely to result in significant functional compromise. Although T4 tumors are typically not suitable for TORS alone, hybrid approaches that combine robotic and open surgical techniques have shown promise. TORS has been applied across a variety of tumor sites, including the palatine tonsil, glossopharyngeal sulcus, base of tongue, pharyngeal wall, soft palate, and, in select cases, the larynx and parapharyngeal space [10]. In clinical practice, TORS is used in primary surgical resections, secondary procedures following radiation therapy, and increasingly in salvage surgeries for recurrent tumors in previously treated areas, with salvage surgery now representing the most common indication (38%) [10]. The expansion of indications has also led to a growing need for reconstruction, especially in cases involving larger resections or exposure of critical structures like bone or the carotid sheath [11,12]. Reconstruction following TORS ranges from secondary intention healing for smaller defects to local or free flap reconstruction for more extensive defects. Tumor size remains one of the strongest predictors for reconstruction, with T3 and T4 tumors often requiring advanced techniques. Additionally, patient-specific factors such as history of radiation, comorbidities (e.g., diabetes, cardiovascular disease, and smoking history), and anatomical complexity play a significant role in both the surgical approach and expected outcomes. As such, reconstruction in TORS requires meticulous attention to preserving function, restoring anatomy, and minimizing postoperative complications. Longfield et al. proposed a helpful algorithmic framework that incorporates tumor location, prior treatments, and patient comorbidities to guide reconstructive planning [12].

### 2.2. Comparison of Traditional Surgical Approaches with TORS

Before TORS, aggressive surgical techniques like lip-splitting mandibulotomy and pharyngotomy were commonly used to treat OPC. While effective in providing tumor access, these approaches resulted in significant morbidity, including extensive scarring, mandibular malunion, osteoradionecrosis, and long-term dysphagia, often leading to feeding tube dependence. These complications prolonged recovery and significantly compromised patients’ quality of life [3].

In contrast, TORS provides a less invasive alternative that enhances precision in resection and flap inset through robotic visualization, with studies showing fewer complications and comparable oncologic outcomes [13]. For instance, Chan et al. demonstrated that TORS resulted in fewer complications compared to traditional methods, even for complex resections like total laryngopharyngectomy [14]. This was especially true for hypopharyngeal cancer cases, where transoral pharyngo-jejunal anastomosis was performed using TORS with free jejunal flaps [14]. Similarly, White et al. compared salvage surgery outcomes between traditional open approaches and TORS for oropharyngeal SCC, showing that TORS led to lower rates of tracheostomy and feeding tube dependence, reduced operative times, and improved recurrence-free survival in salvage cases [15]. For advanced OPC, TORS with radial forearm free flap (RFFF) reconstruction significantly reduced hospital stays while maintaining similar operative times, margin status, and postoperative outcomes, making it a safe, effective, and cost-saving alternative to the more invasive mandibulotomy approach [16].

A recent systematic review analyzed nearly 4000 patients treated with primary TORS for OPSCC, the majority of whom were HPV-positive. While TORS showed good oncologic outcomes, it did not conclusively improve survival or functional results over chemoradiotherapy, though it shows promise for HPV-negative cases [17]. A retrospective study by Porcuna et al. compared salvage TORS with free flap reconstruction to open surgery in stage III-IV OPSCC. The study found that TORS resulted in shorter surgical times, fewer complications, and reduced feeding tube use, while maintaining similar survival rates, further highlighting its ability to reduce morbidity in salvage cases [18].

TORS is especially advantageous for anatomically complex regions that are challenging to access, such as the area between the uvula and the epiglottis. In these cases, TORS enables greater control in flap placement, allowing surgeons to reach deep-seated areas that are otherwise difficult to access. When performing TORS-based reconstructions, key considerations include the preservation of the velopharyngeal sphincter and creating a watertight seal between the pharynx and neck to mitigate the risk of fistulas [13]. Patients who undergo TORS with free flap microsurgery experience synergistic benefits from reduced invasiveness, optimal vascular coverage, and fewer complications such as fistulas, which collectively result in significantly shorter hospital stays [19].

### 2.3. Reconstructive Classification and Techniques in TORS

Reconstruction in TORS often involves both local and free flap transfers. To guide the reconstructive approach, de Almeida et al. classified oropharyngeal defects into four classes based on size and complexity [20]. Adverse features included carotid artery exposure, oropharynx–neck communication, and resection of more than 50% of the soft palate. Class I defects involved a single oropharyngeal subsite with no adverse features, while Class II included multiple subsites without adverse features. Class III involved one subsite with at least one adverse feature, and Class IV included multiple subsites with at least one adverse feature. Class I and II defects typically healed by secondary intention, primary closure, or local flaps. Common reconstructions for these classes included velopharyngoplasties or local flaps. However, Class III and IV defects generally required more advanced reconstructions, such as regional or free flaps [20]. For advanced tumors, particularly in salvage cases, free flap techniques such as the radial forearm free flap are commonly used. A systematic review of 260 patients found that RFFF was the most frequently used flap, with overall favorable outcomes despite some complications, such as fistulas and infections [21]. In general, any thin, pliable fasciocutaneous free flap can be utilized for reconstruction, with alternatives to the RFFF including ulnar forearm flap, medial sural artery perforator flap and lateral arm flap. Minimizing bulk is key to optimizing functional reconstruction, as excess bulk can lead to difficulty with decannulation and swallowing dysfunction for oropharyngeal reconstructions.

Robotic assistance has further expanded the precision of flap inset, particularly in microsurgical procedures. It enhances the accuracy of microvascular anastomoses and flap placement, offering high success rates with fewer complications compared to manual methods [22]. Haymerle et al. reported on the use of TORS to perform free flap inset in six patients with primary or recurrent oropharyngeal squamous cell carcinoma [23]. The flaps were used to reconstruct defects following chemoradiation, prevent velopharyngeal insufficiency, and address large defects. Although the procedure was technically challenging, it was successfully completed using the da Vinci system (Intuitive Surgical, Sunnyvale, CA, USA) without any significant complications. The average surgery duration was 531 min, and hospital stays ranged from 13 to 52 days, with favorable recovery in terms of swallowing function [23]. However, the inset and microvascular anastomoses do not necessarily require the assistance of a robot as they can be performed through a combination of transoral and transcervical approaches. If not included as part of the resection, a small lateral pharyngotomy provides good access to the oropharynx.

A study of 50 patients undergoing free flap reconstruction after TORS for complex pharyngeal demonstrated low complication rates and satisfactory functional outcomes [10]. However, the learning curve was notable, with two cases converted to conventional surgery [10]. Asairinachan et al. discussed the utility of the facial artery musculomucosal (FAMM) flap in salvage TORS reconstructions, particularly for lateral pharyngeal wall defects in irradiated fields. Local flaps were favored over free flaps in these cases to minimize complications, with good outcomes in terms of flap and donor site healing, as well as swallowing and speech function [24]. Another study in 2021 explored the use of the submental artery island flap (SAIF) for reconstruction following TORS in two patients with recurrent oropharyngeal tumors post-chemoradiation [25]. Both cases had successful resections and reconstruction, with no bleeding, fistula formation, flap loss, or donor site complications [25]. The SAIF proved to be an effective reconstructive option for TORS defects, with the flap inset assisted by robotic surgery [25].

Salvage surgeries present unique challenges due to prior radiation and larger defects. TORS has proven effective in these cases, with reconstruction critical for functional and oncologic outcomes. Paleri et al. evaluated TORS in 26 patients with residual and recurrent oropharyngeal cancer, with 21 undergoing TORS and others utilizing robotic-assisted free flap inset. The study demonstrated comparable outcomes to open surgery while offering the benefits of en bloc resection and advanced imaging without the need for mandibulotomy [6].

### 2.4. Oncologic and Functional Outcomes in TORS-Based Reconstruction

In salvage cases where tumors have recurred or patients have received prior radiation, TORS presents a viable alternative to traditional open surgery, offering comparable oncologic outcomes. Porcuna et al. evaluated TORS in a predominantly HPV-negative population and found it to be a safe and effective option for locally advanced or salvage cases, even in non-HPV-associated cancers [26]. Furthermore, TORS can be safely utilized in high-risk cases where the retropharyngeal internal carotid artery is at risk of injury. Careful TORS-assisted resection, combined with free flap reconstruction, has been shown to ensure oncologic safety while protecting vital vasculature, challenging previously held contraindications for using TORS in such scenarios [27].

Functional recovery, particularly regarding swallowing and speech, remains a central focus in TORS reconstructions. Charters et al. conducted a case series on patients undergoing TORS for recurrent or advanced OPC and found that, although swallowing function initially declined post-surgery, most patients regained baseline swallowing and speech function within six months [28]. Long-term quality-of-life outcomes also appear promising. D’Andrea reported that patients who underwent TORS for salvage surgery experienced improvements in both quality of life and swallowing function, with reduced tracheostomy requirements [29]. Similarly, Williamson et al. found that TORS reconstructions, particularly those involving free flaps, resulted in favorable functional and oncologic outcomes, even in salvage settings [30].

## 3. Case Report

### 3.1. Patient Presentation and Surgical Background

**Figure 1:**
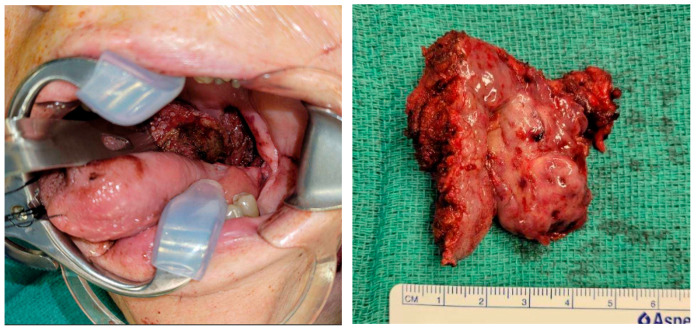
TORS-assisted oropharyngectomy with resection specimen.

**Figure 2:**
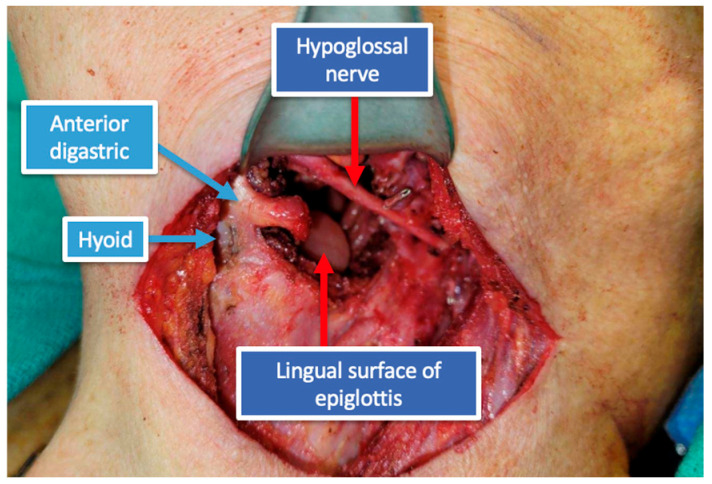
Additional access via a small lateral pharyngotomy. The lingual surface of the epiglottis is visible within the oral cavity.

The patient was a 69-year-old male with recurrent squamous cell carcinoma (SCC) of the oropharynx (Stage I; rcT2, cN1, cM0, p16+) at the base of the left tongue and prior history of chemoradiation. TORS resection was indicated for excision of the recurrent tumor (Figure 1), which involved both the left lateral pharyngeal wall and the base of the tongue. TORS was selected for tumor resection given the patient’s history of prior chemoradiation, the favorable location of the recurrence at the base of tongue and lateral pharyngeal wall, and the availability of robotic expertise at our institution. To address the anticipated defect, the reconstructive plan included a lateral arm free flap to reconstruct the oropharyngeal region, utilizing the superior thyroid artery and a branch of the internal jugular vein as recipient vessels (Figure 2).

### 3.2. Flap Design and Harvest

**Figure 3:**
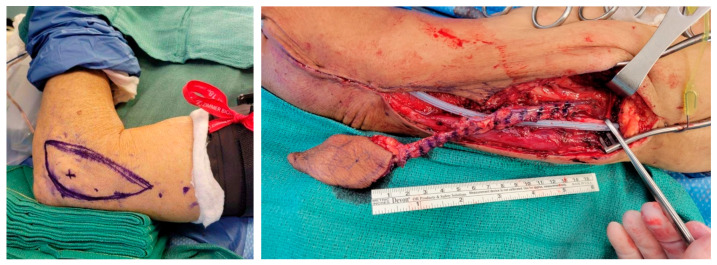
Extended lateral arm free flap.

A lateral arm free flap was selected due to its thin, pliable contour and reliable vascular supply (Figure 3). The skin paddle was marked, centered over the lateral epicondyle of the left arm, and measured 5 cm by 10 cm, determined based on a pinch test to allow for primary closure. A tourniquet was applied following venous exsanguination by arm elevation, and a vertical incision was made over the lateral border of the humerus. Dissection proceeded from distal to proximal in the suprafascial plane while preserving the radial collateral ligament at the elbow.

The posterior radial collateral artery (PRCA) and veins were carefully isolated in the septum between the brachialis and triceps muscles. A 4 cm × 4 cm segment of the triceps muscle was harvested as a pedicled flap to provide additional bulk and stability in the floor of mouth reconstruction. The vascular pedicle was dissected up to its takeoff behind the humerus. This segment was harvested with meticulous care to avoid disruption of nearby neurovascular structures. The radial nerve was identified and protected.

In the neck, the superior thyroid artery and a branch of the internal jugular vein were prepared as recipient vessels. The lateral arm free flap was rendered ischemic, transferred to the oropharyngeal defect, and positioned for inset followed by microsurgical anastomosis.

### 3.3. Flap Inset Technique and Microsurgical Anastomosis

**Figure 4:**
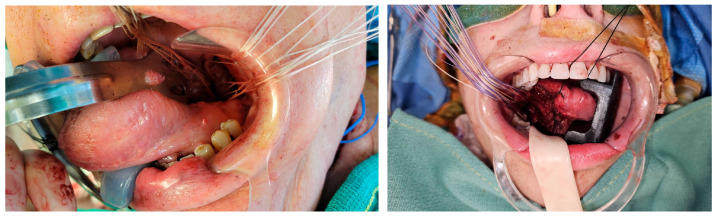
The flap inset is achieved through a combination of transoral and transcervical approaches, with sutures strategically placed and “parachuted” into position. This technique addresses the challenge of limited visualization once the flap is seated, allowing for precise alignment and secure closure in anatomically difficult regions.

Robotic-assisted tumor resection presented significant technical challenges for flap inset due to limited visualization (Figure 4). The lateral arm pedicle was first passed carefully into the neck to the level of the recipient vessels utilizing a penrose drain. Next, attention was turned to inset. The narrow, deep oropharyngeal cavity required careful pre-placement of sutures along the medial aspect of the defect, which were parachuted into position to ensure optimal alignment. Limited direct access further complicated visualization, making precise suturing and flap positioning exceptionally demanding.

After the completion of the flap inset, microsurgical anastomosis was performed, starting with the artery, which was anastomosed using interrupted 9–0 nylon sutures in a 0–180° pattern. The dominant vein of the flap was anastomosed to the internal jugular vein branch using a 3.0 mm mechanical coupler. Following completion of the anastomoses, the Acland clamps were released, and the flap exhibited excellent dermal bleeding and Doppler signals, confirming adequate arterial inflow and venous outflow.

A pedicled segment of the triceps muscle was then inset into the lateral floor of the mouth to provide additional structural support and bolster the primary reconstruction.

### 3.4. Postoperative Course

**Figure 5:**
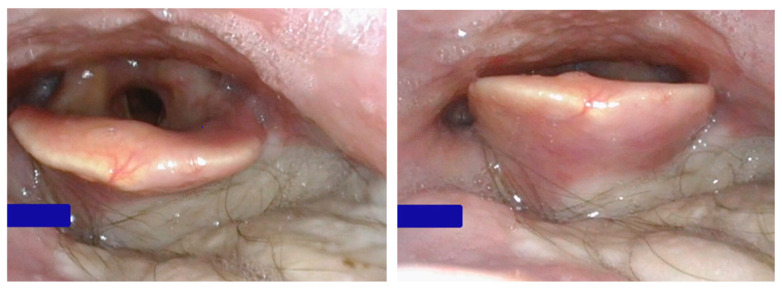
Postoperative view from a nasal laryngoscopy, showing the flap near the epiglottis, well-integrated healing and hair growth.

The patient had an uncomplicated postoperative recovery and was discharged one week after surgery. At his most recent follow-up, the patient reported no fever, chills, pain, nausea, vomiting, or other concerning symptoms. A modified barium swallow (MBS) study at 3 weeks post-op confirmed the integrity of the soft tissue reconstruction (Figure 5), with no evidence of leakage or complications at the flap site.

On physical examination, the neck incision was well-healed without erythema, delayed healing, or signs of infection. Speech was assessed as fully intelligible, indicating favorable functional recovery. The donor site on the left arm also healed without complication, showing no signs of infection, dehiscence, or other issues. No further restrictions to activity were indicated. The patient was cleared to advance his diet under the guidance of speech pathology and was permitted to resume brushing his teeth.

## 4. Discussion

The application of TORS in head and neck reconstruction represents a meaningful advancement in surgical oncology, enabling minimally invasive tumor ablation alongside increasingly sophisticated functional reconstruction. In select cases—particularly salvage scenarios—TORS has demonstrated comparable oncologic control to traditional open approaches, with the added benefits of reduced morbidity and shorter recovery time [18]. Despite these advantages, its incorporation into reconstructive algorithms remains inconsistent across institutions, reflecting a lack of standardized protocols and variability in surgical expertise [19].

Patient selection, tumor characteristics, and timing remain critical factors in determining candidacy for TORS. While TORS is generally suitable for T1–T3 tumors, more advanced cases may necessitate hybrid approaches that combine robotic and open techniques. Reconstructive decision-making must also consider defect location, history of radiation therapy, and patient comorbidities [12]. Classification systems such as the one proposed by de Almeida et al. offer useful frameworks, though they are not yet widely adopted, and few studies have directly compared reconstructive strategies [1].

Although TORS offers significant advantages for appropriately selected patients, it is not universally applicable. The American Society of Clinical Oncology (ASCO) guidelines specify that TORS is FDA-approved for T1 and T2 oropharyngeal tumors but generally not recommended for T4 tumors or those requiring extensive resection of the soft palate due to risks of functional compromise [31]. It is also contraindicated in cases with radiographic or clinical evidence of extranodal extension, matted nodes, or parapharyngeal fat invasion [32]. Anatomic constraints such as trismus, a narrow mandibular arch, or prominent mandibular tori may also preclude safe transoral access [31]. In these scenarios, traditional open surgery remains essential for achieving oncologic and functional goals.

Even when patients meet clinical criteria, institutional factors can limit access to TORS. Many cancer centers do not have robotic platforms or adequately trained surgical teams, creating disparities in access to care. Consequently, open surgery continues to play a vital role, not just when TORS is contraindicated, but also when it is simply unavailable.

Our presented case illustrates successful use of the lateral arm flap in a salvage TORS setting. Although the radial forearm free flap is more commonly employed, this case highlights how flap selection must be tailored based on defect size, required pliability, donor site considerations, and intraoperative access. Alternative fasciocutaneous flaps, including the lateral arm, offer distinct advantages and remain underutilized in many institutions.

Despite increasing adoption, TORS-based reconstruction carries limitations. The steep learning curve, extended operative times, high equipment costs, and logistical demands remain barriers to widespread use [19,23]. Patient anatomy and tumor invasion patterns may require intraoperative flexibility or conversion to open procedures. Furthermore, while many studies demonstrate strong outcomes, complication rates such as flap failure, necrosis, infections, and fistulas have been shown to be similar to those of non-TORS approaches, emphasizing that TORS offers at least comparable, but not necessarily superior, safety [16,33,34]. These findings reinforce the need for rigorous patient selection and coordinated multidisciplinary care. ASCO guidelines support TORS for HPV-positive OPSCC when paired with appropriate neck dissection and individualized planning [31].

Intraoperative and postoperative complication management remains a critical pillar of TORS implementation. Bleeding, reported in 8.1% to 15.2% of cases, requires careful hemostasis and may call for prophylactic transcervical arterial ligation in high-risk patients [31,35]. Dysphagia is commonly encountered but can often be mitigated with early initiation of swallowing therapy. Nerve injuries involving the hypoglossal and lingual nerves can be minimized through meticulous dissection and, when possible, intraoperative monitoring [35]. Larger defects may require free flap reconstruction to restore speech, swallowing, and airway integrity [21,32]. Tracheostomy or gastrostomy may be temporarily required for airway and nutritional support, and their use should be guided by individualized assessment, as emphasized in ASCO guidelines [31].

Looking forward, the field of robotic-assisted reconstruction continues to evolve. Hybrid techniques, such as supracricoid partial laryngectomy with cricohyoidoepiglottopexy (SCPL-CHEP), illustrate how combining transoral and transcervical access may expand oncologic resection options while preserving function [36]. Additionally, pediatric applications such as robotic-assisted cleft palate reconstruction underscore the technology’s versatility in anatomically challenging scenarios [37]. Nevertheless, there remains a pressing need for well-designed, prospective, and case–controlled studies to clarify optimal indications, refine flap selection algorithms, and establish standardized outcome metrics, including tracheostomy timing and long-term functional recovery.

In summary, TORS represents a powerful and evolving tool for head and neck reconstruction, offering precision, minimally invasive access and increasingly reliable reconstructive options. However, its use must be balanced with awareness of technical limitations, resource constraints, and patient-specific considerations. Continued advancements in training, access, and evidence generation will be key to maximizing its impact in complex oncologic care.

## 5. Conclusions

TORS has redefined head and neck reconstruction by providing a minimally invasive alternative to traditional open approaches, particularly for oropharyngeal cancer. While TORS reconstruction provides significant advantages in precision and functional outcomes, its complexity and technical challenges underscore the need for further studies and standardized guidelines.

## Data Availability

No data were generated or analyzed in the preparation of this manuscript.
